# Reconfiguring agency under platform governance: Baby Boomer and Generation X singers negotiating identity, algorithms, and monetization

**DOI:** 10.3389/fsoc.2026.1787838

**Published:** 2026-04-07

**Authors:** Ashanti Hastuti, Suko Widodo, Fendy Suhariadi

**Affiliations:** Development Human Resources, Airlangga University, Surabaya, East Java, Indonesia

**Keywords:** algorithmic governance, copyright, cultural labor, generational identity, global south, music industry, platformization, structuration

## Abstract

**Introduction:**

Digital platforms have reconfigured the organization of music markets through algorithmic curation, metricized visibility, and increasingly formalized copyright regimes. Yet scholarship often centers digital-native artists, leaving less understood how senior musicians negotiate platformization in Global South contexts. This article examines how Indonesian Baby Boomer (1940–1964) and Generation X (1965–1980) singers enact agency within the platform music economy by negotiating generational/professional identity, algorithmic logics, and income-making practices.

**Methods:**

This qualitative study draws on narrative-biographical interviews with 12 informants, including senior singers and key industry stakeholders such as managers, label/publisher representatives, collective management bodies, and aggregators. The interviews were triangulated with public digital traces and policy documents and analyzed thematically using Atlas.ti.

**Results:**

The findings show that senior artists do not experience a linear decline of agency. Instead, agency is reconfigured into layered strategic, practical, and reflexive capacities operating across the structuration modalities of signification, domination, and legitimation. Metrics become a new language of success and decision-making, while control over resources expands through the consolidation of official channels as a digital home and the outsourcing of platform labor to micro-teams and family members. Copyright compliance is internalized as compliance by design, shaping content formats and channel choices alongside demands for more transparent rights governance. These dynamics are intertwined with Adaptation–Aggregation–Arbitrage strategies that stabilize and diversify income across streaming, advertising, licensing, and performance.

**Discussion:**

The study offers a generationally sensitive account of cultural labor under platform governance and highlights implications for industry policy and capacity-building programs in platformized music economies.

## Introduction

Digital transformation over the past two decades has radically reshaped how music is produced, distributed, monetized, and understood. The shift from physical media such as vinyl records, cassettes, and CDs to online listening services funded by subscriptions and advertising has made digital platforms the core infrastructure of the contemporary music economy (Guo, 2023; Pitts, 2015). Streaming, video-sharing, and short-form content now function as key promotional channels and as primary arenas where economic and symbolic value circulates. Industry rationality has moved toward data- and algorithm-driven logics: play counts, watch time, saves, and playlist placement are interpreted as indicators of quality, legitimacy, and bargaining power in commercial partnerships (Falkowski-Gilski and Uhl, 2020; Hilbert, 2020; Hracs and Webster, 2021). In this landscape, artists are expected to manage digital presence, official channels, and cross-platform content portfolios rather than relying on labels and physical distribution alone.

In Indonesia, these dynamics unfold within a distinctive configuration. Recorded-music revenues are now led by streaming, with a mix of paid and ad-supported models in a context where the share of paid subscribers remains relatively low. YouTube, Spotify, TikTok, and similar services have become central nodes connecting artists, audiences, and advertisers, while copyright and collective licensing regimes—through LMK/LMKN and operators such as WAMI—remain key pillars of legitimacy and royalty distribution. Distribution value chains that once depended on producers, distributors, and retail stores have been replaced by direct or semi-direct pathways to digital platforms via aggregators. Automated content identification and rights-claim systems make content-format design and copyright compliance integral to artists’ strategies. Indonesia’s platform-based music economy therefore displays dual tensions: tensions between newly accessible distribution channels and dependence on platform algorithms and policies; tensions between global reach and monetization constraints in a market with limited purchasing power.

Generational categories are global heuristics, but their meanings are locally shaped. In Indonesia, Baby Boomers and Generation X musicians built careers during the dominance of radio/television gatekeeping, cassette and CD economies, and the professional circuits of the late New Order and Reformasi eras. This history matters for platform adaptation: reputations, catalog ownership patterns, and norms of professionalism (rehearsal discipline, studio hierarchies, and label-mediated distribution) formed under earlier infrastructures and are carried, selectively, into platform-based work routines.

Access to digital tools is also uneven. While smartphones and social media are widespread, unequal income distribution, differences in urban–rural connectivity, and the cost of sustained content production (devices, editing, ads, and reliable data plans) mean that “digital-native” creators do not automatically enjoy equal access to visibility or monetization. For senior artists, these constraints often translate into practical arrangements such as partnering with aggregators, labels/publishers, or delegating platform labour to micro-teams and family members.

Gendered expectations may further shape digital participation. As a Muslim-majority country with diverse local moral economies, Indonesia’s norms of public self-presentation can influence how older artists, especially women, navigate visibility, audience interaction, and reputational risk online. This study does not treat gender as a primary analytic variable, but it acknowledges gender as a relevant contextual layer that can condition how platform work (posting frequency, livestreaming, comment moderation) is distributed within teams and households.

Finally, platform use takes place under local regulatory and governance arrangements. Beyond platform community guidelines, Indonesia regulates online services through electronic system provider registration and can impose content restrictions or administrative sanctions that affect platform features and stability. At the time of writing, major music distribution platforms (e.g., YouTube and Spotify) remain broadly accessible, but policy enforcement can be uneven and may include temporary administrative actions affecting certain platform services or features. Such regulatory uncertainty reinforces multi-platform strategies and the importance of rights compliance and documentation when artists manage “official channels” and cross-post content across services.

This structural change is often discussed through the lens of digital-native generations, while the experiences and strategies of generations whose careers began in the analog era receive less sustained attention. Baby Boomer singers (approximately born 1940–1964) and Generation X singers (approximately born 1965–1980), who built reputations in the cassette, radio, and television era, now need to renegotiate their positions in a digital space defined by measurement, speed, and intense competition. They face shifting benchmarks of success, moving from “platinum per copies sold” toward complex platform metrics, while confronting new vulnerabilities related to rights claims and volatile digital income. A central question concerns how their agency—as actors with legacy catalogs, cross-generational reputations, and long professional experience—is realized within a platform music economy organized by algorithms, policies, and rights institutions. More specifically, the question concerns how they negotiate professional and generational identities, respond to algorithmic logics, and reorganize monetization strategies while maintaining artistic integrity.

To date, public discourse about senior artists in the digital era often oscillates between two poles: lag narratives that emphasize digital literacy gaps and nostalgia narratives that celebrate legendary status without examining concrete adaptation practices. These frames obscure the strategic, practical, and reflexive dimensions of older singers’ agency. This generation works to leverage and negotiate the new industry structure. This gap limits the knowledge base needed for industrial policy design and for capacity-building programs attentive to generational realities. A closer reading is needed of how Baby Boomer and Generation X singers in Indonesia manage official channels as a “digital home,” outsource promotional functions, negotiate with labels, aggregators, and LMK institutions, and arrange income portfolios that now draw from combinations of streaming, advertising, public licensing, and offline and online performances. In other words, the research problem concerns how their agency is structured and enacted in everyday practice.

This study addresses these issues by focusing on the agency of Baby Boomer and Generation X singers within Indonesia’s platform music economy through the lenses of structuration theory, prosumption, McDonaldization, and the AAA framework (Adaptation, Aggregation, Arbitrage). Narrative-biographical interviews with cross-generational singers and industry stakeholders (managers, labels/publishers, LMK/LMKN, aggregators) are combined with analyses of content traces and engagement metrics on digital platforms. The study maps how identity, algorithms, and monetization are negotiated in tandem. A structuration perspective helps trace the reciprocal interplay between artists’ actions and structural modalities—meaning, control over resources, and legitimacy—that mediate decisions in production, distribution, and promotion (Giddens, 1984, 1990; Huong and Duc, 2023; Saner et al., 2020). Prosumption and McDonaldization illuminate platform rationalities that prioritize efficiency, calculability, predictability, and control, which push artistic work toward measurable procedures (Ritzer, 2019; Ritzer and Stepnisky, 2022; Sampean, 2026). The AAA framework provides language for interpreting adaptation decisions related to segments and formats, aggregation of processes and assets for scale, and arbitrage across channels and markets when differences in value structures arise (Ghemawat, 2003, 2007, 2018).

More specifically, the article proposes a three-dimensional reading of senior singers’ agency. First, strategic agency refers to the capacity to formulate platform-relevant goals (reach, digital reputation, income sustainability), calculate contractual and rights-claim risks, and choose partnership models—label-dependent, hybrid, or more independent via aggregators—that reduce harmful long-term lock-in. Second, practical agency refers to the ability to operate and/or organize technical functions (home production, official-channel management, release calendar management, community orchestration), including cases where functions are distributed to teams or family members. Third, reflexive agency refers to the skill of interpreting cross-channel metrics, evaluating content experiments, and recalibrating career orientations and public image in response to data feedback and audience reactions. These three dimensions are treated as concrete forms of ongoing negotiation among identity, algorithms, and monetization within a structuration framework.

Prior studies on music industry digitalization have documented platformization, metricized visibility, and new forms of cultural labour in creator economies (Guo, 2023; Hracs and Webster, 2021; Pitts, 2015; Webster, 1991). To situate the present findings in sociological scholarship, we draw on three complementary bodies of literature. First, Becker’s Art Worlds framework conceptualizes cultural production as collective action organized through cooperative networks, conventions, and a division of labour among artists, intermediaries, and institutions. This is directly relevant to platform-era arrangements in which micro-teams, aggregators, labels/publishers, and collective management bodies co-produce both visibility and monetization pathways (Becker, 1982).

Second, Bourdieu’s theory of taste and the field of cultural production clarifies how legitimacy and audience segmentation are structured through symbolic capital and struggles over recognition. Under platform governance, algorithmic curation and metricized attention can be approached as mechanisms that reshape “taste publics,” thereby re-stratifying the circulation of heritage repertoires and contemporary releases (Bourdieu, 1991, 1993, 1996; Collyer et al., 2015; Gilleard, 2020; Go, 2013). Third, scholarship in the musicology of record production emphasizes that recording technologies and studio practices shape musical meaning, performance decisions, and professional roles. This literature provides a vocabulary for interpreting how “compliance-by-design,” arrangement choices, and the redistribution of technical/aesthetic authority operate within platformized production contexts (Bourbon and Zagorski-Thomas, 2020; Homan, 2022; Zagorski-Thomas, 2014, 2016; Zagorski-Thomas and Frith, 2020).

Literature on creators and prosumers further emphasizes the role of users-as-producers in shaping content ecosystems, while McDonaldization research shows how principles of efficiency, calculability, predictability, and control permeate cultural sectors, including music (Ritzer, 2011, 2019; Ritzer and Stepnisky, 2022). Systematic work linking the agency of Baby Boomer and Generation X singers in Global South contexts such as Indonesia to specific institutional configurations—LMK/LMKN, the role of aggregators, and collective licensing practices, remains limited (Hastuti et al., 2024). Explicit integration of structuration theory, McDonaldization, and the AAA framework into a single analytic model also remains relatively rare.

Building on these gaps, the article aims to (i) unpack how the agency of Baby Boomer and Generation X singers in Indonesia is realized within a platform music economy shaped by algorithms, metrics, and copyright regimes; (ii) explain how they negotiate generational and professional identities amid pressures of rationalization and content homogenization; and (iii) identify the adaptation, aggregation, and arbitrage strategies they use to stabilize and expand income sources. The article’s contribution lies in its focus on older generations within a Global South platform-music ecosystem, its effort to connect structuration theory, McDonaldization, and AAA within a layered model of agency (strategic–practical–reflexive), and its empirical account of how official channels, rights systems, and data are managed by artists across generations. The study is expected to deepen understanding of creative agency in platform economies and to offer conceptual and practical foundations for industry policy and capacity-building programs that are responsive to generational dynamics.

## Methods

The study uses a qualitative design with a narrative–biographical approach and thematic analysis. It focuses on Indonesian singers from the Baby Boomer generation (approximately 1940–1964) and Generation X (approximately 1965–1980). These two cohorts built their careers in the analog era and remain active, or have become active again, within the digital music ecosystem. The study involves 12 informants, consisting of Baby Boomer and Generation X singers as the main informants, along with institutional stakeholders, managers, label/publisher representatives, LMK/LMKN representatives, and aggregators as supporting informants. Informants were selected purposively using the following criteria: having a recorded catalog previously released in physical formats, having distributed works through digital platforms (DSPs, YouTube, and similar services), and showing a certain level of engagement on social media or official channels.

Methodologically, the study is positioned as ethnographically informed research. It integrates narrative–biographical interviews with non-participant observation of publicly available digital traces from participants’ official channels and related social media. The trace materials were used to contextualize reported practices, map platform routines (e.g., release cadence and content formatting), and corroborate interview-based interpretations of platform governance and monetization constraints.

Interlocutor recruitment followed purposive, concept-led criteria. We engaged singers who (a) built their careers prior to platform-dominant distribution, (b) maintain recorded catalogues now circulated via digital services, and (c) currently undertake platform-facing work, independently or with the support of micro-teams, related to promotion and monetization. The study is not oriented to statistical representativeness; no robust population register exists for estimating the proportion of older singers participating in Indonesia’s platform economy. The analysis therefore advances analytic generalization by clarifying the mechanisms and conditions through which agency is negotiated under platform governance. Genre and gender are acknowledged as potentially consequential contextual dimensions shaping visibility and audience engagement, yet recruitment was not systematically stratified along these lines. Future research can extend the present findings through genre-focused and gender-sensitive comparative designs.

Ethical safeguards were applied because interviews included potentially sensitive information about contracts, intermediaries, and rights governance. Participants were offered the choice to be identified or anonymized; several preferred anonymity, and the authors applied anonymization consistently across the dataset to protect professional relationships and reduce reputational risk. We use generational labels and participant initials/codes (e.g., Baby Boomer/Gen X) to preserve interpretive traceability while maintaining confidentiality.

Primary data were collected through in-depth narrative–biographical interviews that examined the experience of career transformation from the physical era to the platform era, promotion and distribution strategies, negotiations over contracts and rights, and ways of interpreting metrics and algorithms. Interviews were conducted face-to-face and online, recorded with participants’ consent, and transcribed verbatim. Each participant was assigned a generational code and initials to protect confidentiality. Interview data were triangulated with publicly available digital traces. The ethnographic component of this study is developed through systematic engagement with participants’ publicly accessible platform environments as field materials. For each focal artist, we compiled trace-based notes from official channels and associated social media to document upload cadence, dominant content formats, visible moderation/interaction practices, and cross-posting patterns. These materials were logged as analytic memos and used to contextualize interview narratives, identify routine platform work, and triangulate governance constraints (e.g., claim risks, channel choices, and format design). While this approach does not substitute for prolonged immersion in production sites, it strengthens the study’s capacity to describe everyday platform work *in situ*.

Data analysis proceeded in stages using Atlas.ti. The first stage involved open coding to identify meaning units related to digitalization experiences, survival strategies, and perceptions of algorithms and monetization. The second stage clustered codes into themes organized through three theoretical lenses: structuration (signification, domination, legitimation), prosumption and McDonaldization (efficiency, calculability, predictability, control), and the AAA framework (Adaptation, Aggregation, Arbitrage). The third stage conducted cross-case and cross-generational comparisons to map patterns of strategic, practical, and reflexive agency. Atlas.ti was used to (i) manage the corpus (transcripts, analytic memos, and trace-based notes), (ii) build and iteratively refine a codebook, (iii) link quotations to codes and write within-software memos, (iv) generate code co-occurrence and code-document tables to support comparison across generations and informant types, and (v) maintain an audit trail of interpretive decisions. Trustworthiness was strengthened through source triangulation (singers and stakeholders), data triangulation (interviews and digital traces), peer debriefing, and an audit trail documenting coding and interpretive decisions.

## Results

This section presents the study’s findings in response to three main questions: (i) how the agency of Baby Boomer and Generation X singers in Indonesia is realized within the structure of a platform-based music economy shaped by algorithms, metrics, and copyright regimes; (ii) how they negotiate generational and professional identities under pressures of rationalization and content homogenization; and (iii) how Adaptation, Aggregation, and Arbitrage (AAA) strategies are used to stabilize and expand income sources. The findings are organized by linking cross-case and cross-generational insights across key industry domains, including streaming platforms, video and short-form platforms, copyright systems, intermediaries, and revenue streams.

All excerpts are presented as illustrative instances of recurring patterns identified through cross-case coding; where a mechanism appears across multiple narratives, the text prioritizes analytic synthesis rather than repeated full quotations (see Table 1).

**Table 1:** Cross-generational synthesis of recurring patterns (analytic comparison).

Analytic dimension	Baby Boomer pattern (typical)	Generation X pattern (typical)
Identity positioning & legitimacy	Legacy repertoire and stage professionalism emphasized; heritage/storytelling formats to activate symbolic capital; higher sensitivity to reputational risk in public-facing posts.	Transitional “bridge” identity; combines nostalgia with continuous release and channel professionalism; stronger emphasis on being a rights-conscious business subject.
Platform labour & collaboration (art worlds)	Platform tasks often delegated to family/micro-teams or mediated via labels/publishers; collaboration relies on long-standing industry conventions.	Greater tendency to insource promotion and manage official channels as a “digital home”; collaboration configured through core teams and aggregators.
Metrics & algorithmic sense-making	Initial disorientation; simplified metric readings (views/comments as signs a song is “alive”); decisions often discussed with team.	Metrics more routinely used as decision tools (release timing, boosts, format tests); experimentation framed as iterative learning.
Copyright & governance constraints	Compliance-by-design strongly shapes format choices (e.g., avoiding arrangements that trigger claims); risk management through channel selection.	Compliance paired with demands for reform (transparency/auditable reporting); greater contractual vigilance across multi-use contexts.
Audience interaction & community work	Direct interaction increases in live sessions and greetings, but comment moderation and posting often handled by micro-teams to manage workload and risk.	More active community orchestration across platforms; interaction frequently distributed within the team (responding, moderating, analytics).
Income stabilization (AAA)	Stronger emphasis on catalog and reputation arbitrage (licensing/public performance); adaptation at a pace aligned with capacity and support systems.	More assertive format and channel arbitrage (short-form discovery → official channel conversion); aggregation through SOP-like workflows and repeated templates.

### Realizing agency within the structure of the platform-based music economy

The first set of findings shows that the agency of Baby Boomer and Generation X singers takes the form of layered capacities. These capacities are strategic, practical, and reflexive. They are continually negotiated through three structuration modalities: signification (meaning-making), domination (control over resources), and legitimation (compliance). The shift toward a platform economy positions algorithms, metrics, and copyright regimes as active forces that shape how singers define goals, choose tactics, and evaluate performance.

#### Signification: redefining what counts as “success”

At the level of signification, the shift in “success” markers from physical media to platform metrics appears clearly in multiple data segments. Within the code family “shifts in success indicators,” a Generation X singer stated that past and present measures differ fundamentally:

“*Back then, every 25 thousand copies got you platinum… now it’s confusing… popularity is on radio, streaming, or download; the reports aren’t like they used to be*.” (Interview YNS, Generation X)

This excerpt shows how success, previously anchored in physical certification (platinum per 25,000 copies), is now reframed as dispersed numbers across digital channels. Another set of segments coded as “metrics as decision guides” indicates that platform dashboards have become reference points for post-release decisions, whether a release should be boosted again, given an acoustic version, or left as part of the back catalog. Many singers do not read metrics in technical detail, yet most informants acknowledged that views, streams, comments, and playlist placement have become a new language for interpreting their position in the platform economy. For Baby Boomers, this process often begins with disorientation; they “learn slowly” to read weekly increases in views and incoming comments as signs that a song remains alive in public attention. Algorithms and metrics therefore function as meaning-making devices that define what is understood as “successful,” “rising,” or “declining.”

#### Domination: resource control and the width of agency

At the level of domination, code families on “digital capital,” “social capital,” and “symbolic capital” show that control over resources determines how wide an artist’s room for agency becomes. The code “direct access to DSPs/aggregators” appeared repeatedly in Generation X narratives. One stakeholder summarized the change in distribution architecture:

“*Now people go direct. They produce it and throw it straight to Spotify… or to an aggregator, then to YouTube… in the past there were producers, distributors, agents, stores; once it went digital it went straight to music platforms, straight to the public.*” (Interview AD, Generation X)

This segment was coded under domination because it highlights a shift in control points: technical and organizational capacity to access aggregators and DSPs allows artists (or their management) to take over functions previously dominated by labels and distributors. In promotion, the code cluster “in-house promotion” reinforces the importance of internal digital and social resources. A Generation X singer emphasized that promotion can no longer be outsourced entirely:

“*Now people make music videos on their own YouTube channels… for their own official YouTube channel, and on our socials… promotion has to be handled by our own team, you can’t just sit back*.” (Interview IP, Generation X)

Within a structuration lens, this reflects a shift in domination from external institutions to the artist’s micro-team: whoever controls official channels, a small production setup, and social networks gains greater agency in setting release rhythms and distribution strategy. Reliance on teams and family members also shows how resource control becomes internalized. A Generation X singer who described themselves as not “technological” explained how roles are distributed:

“*Honestly, I’m not the type who gets involved with technology… in the last two years my younger sibling helped… all releases are handled [by the team/manager]… later we look at what the final report means for us*.” (Interview KD, Generation X)

Here, domination does not mean the artist personally controls every tool. It refers to the capacity to organize resources (siblings, managers, teams) so that digital functions operate effectively. In contrast, Baby Boomer accounts coded as “dependence on labels/publishers” and “family support” describe a different distribution of resources: they more often continue working with labels/publishers as rights custodians and traditional gatekeepers while relying on small teams for day-to-day digital operations. Across cases, Atlas.ti output shows that the codes “promotion managed internally” and “official channel as a digital home” appear more frequently in Generation X narratives than in Baby Boomer narratives. This supports the conclusion that this transitional generation tends to insource digital and social capital management to a greater extent.

#### Legitimation: copyright and platform policy as concrete boundaries

At the level of legitimation, code families related to copyright and platform policies function as concrete boundaries for agency. Two frequent codes were “fear of a second master” and “compliance from the design stage.” A Baby Boomer singer explained why they avoid changing arrangements when performing on their own channels:

“*What’s not allowed is when a singer wants to sing their song on their own TikTok, on their own YouTube, but uses a different arrangement. That’s not allowed; we have to be responsible to the composer… so it has to be the same… If you do that, it turns out you’ll create two masters. The first master is the original, then there’s a second master… If you want to create a second master you have to pay a certain amount and ask permission again from the songwriter and arranger*.” (Interview RDS, Baby Boomer)

This excerpt, consistently coded as “second-master risk” and “copyright compliance,” shows how legal norms translate into concrete strategy: the artist chooses an official minus-one track rather than creating a new arrangement that might trigger claims. As noted earlier (Interview RDS, Baby Boomer), adaptation is operationalized through “compliance-by-design”: the artist mobilizes label-provided minus-one assets and short-duration platform formats to sustain visibility while minimizing claim risks. Rather than repeating the quotation, this section treats the case as an instance of format–rights co-optimization within a platformized revenue portfolio.

From these segments, Atlas.ti grouped a pattern coded as “compliance by design.” Singers deliberately manage format (minus-one), channel selection (YouTube versus TikTok), and duration (1 minute) to remain within legal corridors while pursuing reach. For Generation X, similar code families appeared as calls for institutional reform. One informant argued that LMK should remain in place while being rebuilt through digitalization:

“*Basically, LMK needs to be fixed, they should use digital. Keep LMK. I want LMK to stay, but it should be restructured, and the way is through digital. Everyone should be able to see it—how much is mine—make it through an application*…” (Interview VP, Generation X)

This reflects a “digital-first legitimacy” orientation. Compliance here includes an effort to push for new norms that strengthen transparency and accountability. Legitimation therefore operates in two directions: artists follow copyright rules and platform policies while seeking changes through demands for digitalization and traceable rights management.

#### Forms of agency derived from coding patterns

The coding results indicate that the three agency dimensions—strategic, practical, and reflexive—are grounded in recurring transcript patterns rather than abstract theoretical constructs. The code family “strategic agency” contains segments on measurable goal setting (reach, conversion, income), partnership model choices (label-dependent, hybrid, or aggregator-oriented independence), and contract and rights negotiations. AD’s account of direct-to-platform pathways and KD’s discussion of layered contracts for soundtracks and television broadcasts fall within this family, showing awareness of contractual complexity and judgments about when to rely on labels and when to self-manage.

“Practical agency” includes segments on everyday digital literacy, routinized production and distribution, and community orchestration. KD’s account of releases handled with a sibling’s support and IP’s emphasis on team-run promotion, together with Baby Boomer narratives about weekly live karaoke sessions using minus-one tracks, show how daily practical work is shaped by platform affordances. Adjustments such as vertical video formats, short duration, hook-focused structure, and calls to direct audiences to official channels were grouped under the sub-code “format-fit platform,” linking practical work to algorithmic demands.

“Reflexive agency” captures segments that show reinterpretation of experience, metrics, and position within the structure. YNS’s confusion about streaming reports followed by learning about revenue sources; VP’s request for an LMK application; and Baby Boomer accounts of reducing arrangement experimentation after rights claims all indicate double hermeneutics in practice: artists interpret structures (metrics, rules, institutions) and allow their actions to shape structural conditions (such as pushing LMK digitalization).

The agency of Baby Boomer and Generation X singers in the platform music economy is realized through three traceable layers of capacity: strategic capacity to choose release pathways and partners, practical capacity to routinize platform-suited digital work, and reflexive capacity to interpret metrics and advocate for more equitable rights governance. These capacities are continually negotiated within the fields of signification, domination, and legitimation shaped by algorithms, metrics, and copyright regimes. The structure functions as a landscape that encourages singers to design goals, manage opportunities and constraints, and build new routines while sustaining core artistic values and identities.

### Negotiating generational and professional identity

The second research question examines how Baby Boomer and Generation X singers negotiate generational identity and professional identity under pressures of rationalization (efficiency, calculability, predictability, control) and a tendency toward content homogenization in the platform economy. The Atlas.ti coding results indicate that this negotiation goes beyond technical adjustment to technology. It is a reflective process through which singers renegotiate how they are positioned, and how they position themselves, in digital public space. The most salient code families include “generational identity,” “singers’ professionalism,” “format rationalization,” and “preserving rasa.”

#### Baby boomer: “cassette/TV-era artists” and legacy as symbolic capital

Within the Baby Boomer cluster, self-identification as “cassette/TV-era artists” emerges strongly in biographical narratives. They emphasize stage professionalism, disciplined rehearsal, and habitual work routines shaped by the physical recording industry. As they enter the platform era, several senior singers recognize external stereotypes that frame them as “older products” and therefore less attractive to sponsors or younger audiences. This appears in fragments coded as “categorized as an old product by sponsors” and “industry doubts about senior artists.” These narratives, however, do not end with passive acceptance. Under the code family “legacy as symbolic capital,” Baby Boomer singers describe how classic catalogs and extensive stage experience become sources of authority and emotional depth. They redesign self-presentation on digital channels through storytelling formats and intimate performances rather than copying younger artists’ promotional styles.

Empirically, this can be traced through segments that combine older assets with platform formats. One Baby Boomer singer, RDS, describes positioning herself as a carrier of collective memory while participating in the digital arena. She explains that recordings and minus-one tracks from the cassette era remain the basis of her performances on new channels:

“*Lately it’s been hard to find songs… But it’s prepared, because I have dozens of minus-one tracks from my music label… I just press play.”* (Interview RDS, Baby Boomer)

This quotation points to a strategy (using official minus-one tracks) and a continuity of identity: voice, repertoire, and performance “rasa” remain consistent while the distribution medium shifts. In other segments coded as “sharing stories behind songs” and “greeting fans across age groups,” Baby Boomer singers explain that live streams and short uploads are used to narrate the histories of classic songs, interpret lyrics, and address younger listeners who have only recently encountered their work. Generational identity as “cassette-era artists” is rearticulated into a cross-generational narrative that frames them as custodians of musical heritage.

#### Generation X: a transitional position and “digital home” professionalism

Generation X singers occupy a distinctive transitional and adaptive position. They began their careers within radio, cassette, and television ecosystems, and they remained active as the internet, YouTube, and digital streaming consolidated. Code families such as “analog–digital transition” and “nostalgia and adaptation” appear consistently in their narratives. A Generation X singer, YNS, describes the changing indicators of success:

“*Back then, every 25 thousand copies got you platinum… now it’s confusing… popularity is on radio, streaming, or download; the reports aren’t like they used to be.” (Interview YNS, Generation X)*

This segment, placed in the code family “generational shifts in success indicators,” shows how professional identity shaped by platinum-era logic must be renegotiated as success metrics fragment into streams, views, downloads, and engagement. The change reaches the level of self-understanding as a “successful singer.”

Beyond metric fragmentation, Generation X narratives also show a shift toward internally managed promotion and experimentation with platform formats. “Official channels” are constructed as a professional “digital home,” where musical assets, visual identity, and audience interaction are consolidated and managed through coordinated routines rather than *ad hoc* posting. Across Generation X narratives, professional identity is increasingly anchored in maintaining an “official channel” as a digital home and in delegating platform labour to micro-teams or family members (Interviews IP; KD, Gen X). This supports the article’s central claim that senior singers’ agency is frequently exercised through orchestration, coordinating resources, routines, and collaborators—rather than through individual technical mastery alone. Interpreted through Becker’s Art Worlds perspective, this pattern indicates a reconfiguration of the division of labour in cultural production: channel governance, promotional coordination, and intermediary work become constitutive components of the production network rather than peripheral “marketing” tasks (Becker, 1982).

This orchestration is often sustained through intra-family role divisions and micro-team arrangements. The singer maintains a performer identity focused on vocal quality and repertoire choices, while technical and some managerial functions are delegated to close relations. In the coding scheme, this sits at the intersection of “professional identity as performer” and “digital management by an internal team.” Adaptation is framed as organizing support around the core role of singer rather than transforming into a full-time content creator.

#### Rationalization, homogenization, and the boundary work of “preserving rasa”

Pressures of rationalization and content homogenization are captured most clearly in the code family “adjusting to platform format and rhythm.” Singers from both generations recognize that algorithm visibility requires certain routines: regular single releases, teasers, hook-based short clips, and sustained posting frequency. The derived code “preserving rasa” highlights efforts to avoid full absorption into homogenized content patterns. Singers negotiate boundaries between following format expectations and sustaining identity.

Among Baby Boomers, emphasis on “human rasa” and vocal authenticity often emerges in discussions of production technology. RDS, for example, acknowledges the usefulness of technology and minus-one tracks, while consistently elevating interpretation and emotional connection with audiences. These segments were coded as “technology as support, not a replacement for rasa.” Digital tools, including AI-based tools, are treated as instruments for efficiency rather than substitutes for vocal expression or stage improvisation that define their generational style.

Generation X singers, who are more familiar with digital format logic, also resist uniformity. In segments coded “format–identity compromise,” they explain that song duration, structure, and production style may be adjusted for streaming compatibility, while distinctive elements are retained through vocal color, harmonic choices, or lyrics that preserve thematic depth. Their narratives present “working with the algorithm” as a practice that can coexist with maintaining artistic selfhood. In promotional content, several Generation X informants avoid viral trends they consider misaligned with age or persona. They develop reflective, story-driven content that emphasizes long career journeys. Codes such as “not forcing a youthful style” and “a modest senior persona” recur in Generation X transcripts, indicating subtle negotiation between popular culture pressures and identity comfort.

#### Professional identity as business subject and rights-conscious actor

Professional identity negotiation also appears in relationships with industry intermediaries. The code family “singer as a business subject” includes Generation X narratives describing deeper involvement in contractual decisions and rights governance. KD, for example, explains that contracts differ across film soundtracks, soap operas, and television broadcasts, and uses the phrase “layered contracts” to describe the need for vigilance:

“*If… a contract with one label for a film soundtrack…, the contract is different when it’s used for soap operas, TV… now our contracts are layered… there’s a blanket system… it should be implemented*…” (Interview KD, Generation X)

This segment indicates that professional identity now includes a role as a contract-literate actor. They question clauses and revenue-sharing schemes rather than fully deferring to labels. Several informants also describe limiting endorsements or commercial posts on personal channels in order to keep the channel oriented toward music. This pattern was coded as “keeping the channel as a music space,” reflecting an effort to maintain a primary professional identity as singer rather than shifting toward a generalized influencer role.

Legitimation is negotiated toward more equitable arrangements. The code family “pushing LMK digitalization” includes statements such as VP’s:

“*Basically, LMK needs to be fixed, they should use digital. Keep LMK. I want LMK to stay, but it should be restructured, and the way is through digital. Everyone should be able to see it—how much is mine—make it through an application…*” (Interview VP, Generation X)

This excerpt shows that senior singers do not simply comply with copyright regimes; they demand more transparent governance. In terms of professional identity, they present themselves as rights-holders who expect auditable systems rather than passive royalty recipients. This reinforces an image of professionals who understand structural arrangements and act within them.

The empirical evidence for the second question shows that rationalization and content homogenization in the platform economy do not automatically erase generational differences or diminish senior singers’ professionalism. Across the layered codes “generational identity,” “singers’ professionalism,” “format rationalization,” and “preserving rasa,” Baby Boomer and Generation X singers manage the “senior” label as a source of legitimacy and relational closeness. Baby Boomers articulate musical heritage as symbolic capital. Generation X positions itself as an analog–digital bridge with a transitional and adaptive stance. Both generations use platform-compatible routines such as single-by-single releases, short-form content, and posting rhythms. Their narratives describe ongoing efforts to weave these routines into an artistic identity grounded in rasa and professional selfhood. This negotiation enables them to sustain generational and professional identities under platform logics that promote standardization.

## Stabilizing and expanding income

The third research question examines how Baby Boomer and Generation X singers use the AAA strategy (Adaptation, Aggregation, and Arbitrage) to stabilize and expand income sources within the platform economy. Atlas.ti coding shows that all three components recur consistently across the data, although their intensity and forms vary across individuals and generations. The main code families are “format & release-calendar adaptation,” “work and asset standardization,” and “channel–time–rights arbitrage.” Together, they describe how singers treat the platform economy as a space for managing an income portfolio rather than a space for performance alone.

### Adaptation: format, narrative, and release calendar

Within Adaptation, the code families “format adaptation,” “narrative adaptation,” and “release-calendar adaptation” appear in almost every case. Format adaptation is visible in choices to repackage older and newer repertoires to fit the affordances of particular channels. Segments coded as “acoustic/minimal versions” and “intimate live sessions” show how classic songs are carried into formats that align with digital consumption. A Baby Boomer singer explained that she now performs in a simpler yet consistent format, using official minus-one tracks as a base:

“*Lately it’s been hard to find songs… But it’s prepared, because I have dozens of minus-one tracks from my music label… I just press play*.” (Interview RDS, Baby Boomer)

This segment indicates rights compliance (legitimation) and operational–creative adaptation: older repertoires are reactivated through a “live karaoke” format suitable for live streaming and short content, with a lower risk of triggering claims. In the Generation X cluster, format adaptation also appears through vertical video, hook-based cuts, and stage snippets prepared for TikTok and Reels. The code “format-fit platform” groups segments where singers explain why they cut specific sections into short clips designed to draw audiences toward full versions on official channels or digital listening services.

Narrative adaptation emerges strongly in codes such as “stories behind songs” and “reframing classics as heritage.” Baby Boomer singers use live sessions to recount a song’s history, the recording context from earlier periods, and the meaning of lyrics for them personally. This approach does not translate directly into immediate revenue, yet it was coded as “activating rasa and attachment,” which supports streaming stability and stage invitations. Among Generation X singers, adaptive narrative appears in efforts to combine nostalgia with contemporary framing: older songs are reintroduced through slightly updated arrangements, while new releases address themes circulating in social media conversations.

Release-calendar adaptation is captured in codes such as “single-by-single strategy” and “using momentum.” A Generation X stakeholder, AD, described the new distribution architecture that enables this approach:

“*Now people go direct. They produce it and throw it straight to Spotify… or to an aggregator, then to YouTube… in the past there were producers, distributors, agents, stores; once it went digital it went straight to music platforms, straight to the public*.” (Interview AD, Generation X)

Direct access to DSPs and aggregators allows singers to manage release rhythms one track at a time to test market response rather than waiting for a full album cycle. In several cases, the code “testing response before an album” appears when Generation X singers explain that early single metrics—plays and saves—inform decisions to proceed with a second single, revise promotion strategy, or delay album production. This calendar adaptation spreads risk and maintains movement in streaming income flows rather than concentrating expectations on one large release moment.

### Aggregation: SOPs, official-channel consolidation, and stable core teams

The second element, Aggregation, is evident in the code families “production–distribution SOPs,” “official-channel consolidation,” and “mid-term core teams.” Production–distribution SOPs refer to efforts to standardize workflows so scale can increase without a linear rise in workload. Segments in this group describe using project templates in recording software, standardizing file formats (master, instrumental, radio edit), and maintaining orderly folder structures to facilitate uploading across platforms. Informants rarely use the term SOP explicitly, yet descriptions such as “the same format every time,” “files already packaged,” and “just send it to the aggregator” indicate the aggregation of work into repeatable patterns.

The “official-channel consolidation” code family highlights YouTube channels and official social media accounts as a “digital home” that aggregates assets. The “official-channel consolidation” pattern shows YouTube channels and associated social media functioning as aggregation nodes (a “digital home”) for music videos, performance archives, and short-form content. As described earlier (Interviews IP; KD), these routines are commonly sustained through micro-teams and family-based role divisions, enabling continuity of output and interpretive work around metrics without requiring the singer to personally operate every technical component.

This aggregation of functions reduces transaction costs: the singer does not need to re-explain procedures to new parties, and the core team learns the artist’s preferences and platform dynamics cumulatively. In Atlas.ti analysis, “mid-term core team” frequently co-occurs with “stable production rhythm,” suggesting that managing multiple income streams (streaming, live shows, paid content, brand partnerships) depends heavily on team consistency and standardized procedures.

### Arbitrage: costs, time, channels, and rights/catalog

The third element, Arbitrage, shows the widest variation and appears in several code clusters: “cost arbitrage,” “time and momentum arbitrage,” “channel arbitrage,” and “rights and catalog arbitrage.” Cost arbitrage is visible when informants describe moving from large studios to home studios and independent recording. Several Generation X singers noted that affordable recording equipment enables high-quality demos without renting expensive studios, lowering per-song production costs and expanding room for experimentation. The code “home studio as leverage” marks these segments, which relate to income stability through reduced fixed costs and reduced dependence on a single large release to recover production expenses.

Time and momentum arbitrage appears in how singers and stakeholders interpret advertising cycles, release competition, and listening habits. Institutional informants explained that digital income collected through LMK/LMKN and WAMI fluctuates: certain years involve delayed payments that accumulate and are disbursed in large sums, while subsequent years appear lower even when consumption has not dropped sharply. These segments were coded as “digital income volatility” and “dependence on platform cycles.” For artists, awareness of volatility supports time arbitrage through more intensive campaigns during peak periods and cash-buffer planning during downturns. At a micro level, the choice of release day and hour—considering other artists’ release schedules—appears in codes such as “avoiding release collisions” and “choosing peak audience hours.”

Channel arbitrage is most clearly captured in the code family “functional channel division.” Singers and teams position TikTok and Reels as spaces for discovery and closeness, YouTube as an archive and a source of advertising revenue, DSPs as the backbone of audio royalties, and karaoke and live stages as public-licensing arenas. RDS’s differentiation between YouTube and TikTok provides an empirical example:

“*On YouTube we can get hit, the royalties get hit… That’s why our songs have a publisher that’s also someone else. On TikTok it’s safer because it’s only one minute*.” (Interview RDS, Baby Boomer)

This segment was coded as “different channels, different risks” and “TikTok as a safer short-duration space.” The pattern indicates a strategy in which short clips trigger attention and emotional closeness, directing audiences to official channels (YouTube/DSPs) for full consumption that supports monetization. Channel arbitrage here refers to assigning distinct roles to each channel within an exposure–conversion–income pathway.

Rights and catalog arbitrage is particularly salient among Baby Boomers. The code family “reviving older catalogs” and “public licensing as a stable source” groups segments where LMK/LMKN informants explain tariff schemes for karaoke venues, hotels, restaurants, and other public spaces, including a minimum 80% share for rights holders. Senior singers treat classic catalogs as assets that can continue generating revenue through public licensing and synchronization, while newer materials are oriented toward streaming and digital campaigns. Among Generation X singers, rights arbitrage more often appears through combinations of commercial projects—film or television soundtracks with layered contracts—and more experimental independent releases. KD describes this complexity:

“*If… a contract with one label for a film soundtrack…, the contract is different when it’s used for soap operas, TV… now our contracts are layered… there’s a blanket system… it should be implemented…”* (Interview KD, Generation X)

This segment, along with the code “contract calculation and downstream use,” indicates that rights arbitrage involves long-horizon calculations: decisions about which works to assign to labels for broad coverage and upfront advances, and which works to retain for independent management and future cross-channel flexibility.

Across these patterns, the AAA strategy contributes to income stabilization and expansion. Adaptation sustains relevance and prevents channels from becoming inactive, supporting ongoing streams and views rather than dependence on a single major release. Aggregation lowers transaction costs and strengthens a team’s capacity to manage multi-source income portfolios. Arbitrage enables singers to use differences in costs, time cycles, channel characteristics, and rights regimes to build combinations that are more resilient to algorithm changes and advertising cycles.

Cross-generational analysis in Atlas.ti suggests that Generation X tends to be more assertive in format adaptation and channel arbitrage, linked to higher digital literacy and a tendency to insource promotion and community management. Baby Boomers show stronger emphasis on rights arbitrage and reputation, maximizing the value of classic catalogs and stage credibility while adopting adaptation and aggregation at a scale aligned with their capacity. The data indicate a convergence process: Baby Boomers gradually adopt agile digital practices associated with Generation X, while Generation X draws on the consistency of professional practice and the depth of rasa carried by earlier generations.

The findings for the third question show that AAA is realized as everyday micro-decisions about format, channels, release calendars, asset governance, and rights negotiations. Through combinations of Adaptation, Aggregation, and Arbitrage, Baby Boomer and Generation X singers treat the platform economy as a medium for sustaining careers and developing income streams over time.

## Discussion

The platform-based music economy does more than shift distribution media. It reshapes the logic of creative work and the governance of value, influencing how “success” is interpreted, how resources are controlled, and how copyright compliance becomes embedded in everyday decisions. The findings indicate that Baby Boomer and Generation X singers do not occupy a passive position in relation to algorithms, metrics, and rights regimes. They develop layered forms of agency (strategic–practical–reflexive) that are continuously negotiated through the structuration modalities of signification, domination, and legitimation. This pattern extends the view that platforms function as structuring arenas where artists’ actions and platform rules co-produce each other (Giddens, 1984, 1990, 1996; Huong and Duc, 2023; Saner et al., 2020).

Within signification, YNS’s statement (“Back then, every 25 thousand copies got you platinum… now it’s confusing…”) illustrates how formerly stable and centralized indicators of success—such as unit-sales certification—have transformed into fragmented metric configurations across channels (streaming, downloads, radio, views, engagement). This supports the argument that platformization drives a shift toward datafied success, where numbers function as outcomes and as a language that shapes artists’ perceptions of position, quality, and strategic viability. Prior literature has emphasized the dominance of metrics and algorithms as new evaluation regimes in the creative industries, including music (Falkowski-Gilski and Uhl, 2020; Hracs and Webster, 2021; Keith et al., 2022; Thorgersen, 2020). The contribution of this study lies in showing how this metric regime operates along generational lines: Generation X tends to internalize metrics as decision tools (“metrics as decision guides”), while Baby Boomers often move through an initial phase of disorientation before adopting simplified readings of metrics through indicators closer to affective experience, such as weekly rises in views or comments as signs that “the song is still alive.” This suggests that data interpretability involves more than digital literacy. It involves meaning translation, where metrics are converted into artistic experience and work habits shaped in the analog era.

Situated in cultural sociology, these shifts can also be read through Bourdieu’s account of taste and distinction: metrics and algorithmic rankings do not merely measure popularity, they participate in producing hierarchies of attention that can reclassify artists and genres, shaping which publics encounter “heritage” repertoires versus “new” sounds. Generational identity thus operates as symbolic capital that is continually tested and re-valued within algorithmically curated taste infrastructures (Bourdieu, 1991, 1996; Bourdieu et al., 1994; Bourdieu and Wacquant, 1992).

Within domination, the findings show a shift in control centers from older institutions (labels–distributors–stores) toward a more micro configuration: artists’ core teams, aggregators, and official channels as asset nodes. AD’s statement (“Now people go direct…”) captures the transformation from long distribution architectures into direct or semi-direct pathways to platforms. This aligns with platformization literature showing that digital distribution dismantles older value chains while creating dependence on platform policies and new intermediaries such as aggregators (O’Laery, 2023; Thorgersen, 2020; Zhang and Negus, 2024). In this context, domination does not refer to individual technical mastery. It refers to the capacity to organize resources, digital, social, and symbolic to secure operational control. IP’s remark (“promotion has to be handled by our own team, you can’t just sit back”) shows that agency expands when artists insource promotion and configure official channels as a “digital home” that consolidates content, identity, and conversion pathways. KD’s account (“helped by my younger sibling… all releases handled [by the team/manager]”) highlights a collective and distributed form of domination: artists with limited technical confidence can still enact agency through delegation and orchestration within an internal team. These findings challenge the common binary of “digitally literate versus left behind” often applied to senior artists. The more decisive factor is the capacity to build a working system (team, procedures, aggregator access) that enables stable digital labor.

Becker’s notion of Art Worlds is useful here: what appears as an individual artist’s “adaptation” is often the outcome of coordinated work among collaborators (micro-teams, family members, editors, aggregators, labels/publishers, and rights institutions) operating through shared conventions about ‘official’ releases, metadata, and platform routines (Becker, 1982; Mazziotti, 2020; Meyn et al., 2023; Sampean, 2026; Shrestha et al., 2020; Xue, 2022). In Becker’s terms, the relevant unit of analysis is not the individual artist but the cooperative network that makes production possible through shared conventions and a division of labour. In the platform era, conventions of “officialness” (channel governance, metadata discipline, and release formatting) function as coordination devices that stabilize collaboration across teams, aggregators, and rights institutions and, in turn, shape how agency is enacted. This helps explain why IP’s and KD’s accounts foreground delegation and orchestration: agency emerges from the capacity to coordinate roles, routines, and resources within a platformized art world, rather than from individual technical mastery alone.

Record-production scholarship likewise highlights that technical decisions in recording and post-production are inseparable from aesthetic decisions; the rise of home studios and desktop workflows redistributes authority across performers, producers, and engineers (Bourbon and Zagorski-Thomas, 2020; Homan, 2022; Sampean, 2026; Zagorski-Thomas, 2014). These perspectives help explain why delegation and workflow aggregation can expand, rather than simply diminish, senior artists’ practical agency in the platform era.

Within legitimation, the study shows that copyright compliance functions as a strategic variable integrated into content design and channel selection rather than an external constraint alone (Xue, 2022; Yurui et al., 2024). RDS’s narrative about a “second master” and the risk of new arrangements demonstrates how legal norms and platform claim systems drive compliance by design: choosing official minus-one tracks, avoiding certain uploads on YouTube, and using short-duration TikTok formats perceived as safer. Here, copyright regimes and automated content-identification systems provide boundaries and opportunity maps (Xue, 2022; Yurui et al., 2024). This strengthens the view that, in a platform music economy, legitimacy is embedded in infrastructure: rules are institutionalized through LMK/LMKN and enacted through platform system design that automates claims and monetization. VP’s call for LMK digitalization (“make it through an application… everyone can see”) points to a dual direction of legitimation: artists comply with existing rules while demanding governance reform that supports transparency and auditable accountability. Reflexive agency here functions as critique of information asymmetries, focusing on mechanism renewal that aligns with data-intensive platform logics.

From the perspective of prosumption and McDonaldization, the findings confirm that senior singers’ work undergoes procedural rationalization: efficiency through work templates and SOP-like routines; calculability through performance indicators; predictability through repeated content formats (teasers, hooks, periodic releases); and control through algorithmic systems and platform policies governing visibility and claim risk (Duffy and Meisner, 2023; Silahtaroğlu et al., 2021). The study also shows that rationalization does not produce total homogenization. In the second research question, the code “preserving rasa” captures boundary strategies: Baby Boomers treat technology as support rather than a substitute for expression; Generation X develops format–identity compromises, adjusting duration or streaming-friendly structures while maintaining vocal color, harmonic choices, and lyric style. McDonaldization logics therefore do not erase artistic differentiation. They trigger micro-level negotiations over which elements become “rationalized” and which remain core identity markers. This matters because content homogenization emerges as a consequence that depends on artists’ positions, strategies, and capitals rather than an inevitable outcome.

The generational identity negotiations, Baby Boomers as “heritage custodians” and Generation X as an “analog–digital bridge” show that identity functions as a managed resource and as symbolic capital in the digital ecosystem. RDS’s account of label-provided minus-one tracks (“dozens… just press play”) demonstrates how analog-era assets and long-standing relationships (with labels/publishers) can be converted into legitimacy and digital production material. Generation X draws on its transitional position to articulate professional authority (quality standards and experience) and communicative proximity (short content, official channels, in-house promotion). These findings extend generational scholarship in music that often positions Generation X as transitional. This study adds detail by showing that “transitional” includes resource-management strategies: insourcing promotion, experimenting with formats, and consolidating channels as primary digital assets.

Findings for the third research question show that the AAA framework clarifies income-portfolio logics in the platform economy. Adaptation operates at the level of format (vertical content, hook cuts, live karaoke), narrative (stories behind songs, heritage framing), and release calendar (single-by-single) (Ghemawat, 2003, 2018). This aligns with accounts that platforms function as major arenas of economic–symbolic value circulation and require cross-channel content strategies (Guo, 2023; Pitts, 2015). Aggregation shows that income stability is shaped by managerial capacity: production–distribution SOPs, official-channel consolidation, and mid-term core teams that reduce transaction costs. Arbitrage highlights monetization as a practice of working with differences: differences in claim risk (YouTube versus TikTok), channel functions (discovery versus full monetization), production costs (home studios), and rights value (older catalogs for public licensing; newer works for streaming and campaigns). The study adds an important insight that, for senior artists, rights and reputation arbitrage offers distinctive stability: catalogs and stage credibility operate as buffers when algorithms shift or advertising revenue fluctuates.

Theoretically, the article strengthens the proposition that creative agency in platform economies should be understood as layered capacity produced through structuration relations rather than as an individual attribute. Strategic agency appears in partnership choices (dependent, hybrid, aggregator-mediated independence), contract negotiations, and claim-risk calculations. Practical agency appears in routinized digital labor: channel management, team role division, and format adjustment. Reflexive agency appears in metric interpretation, experiment evaluation, and demands for rights-governance reform. By linking structuration to platform rationalization, the article shows that senior artists develop forms of strategic compliance and careful innovation that support career sustainability under algorithmic control. The novelty lies in mapping concrete mechanisms: metrics as a language of meaning, official channels as domination assets, and rights compliance as operational design.

The practical implications of these findings are relevant for industry policy and capacity-building programs. First, capacity strengthening should focus on more than technical content-production training. It should focus on building a work system: core teams, asset SOPs, channel management, and contract literacy, because these factors shape aggregation capacity and stability. Second, rights governance requires transparency compatible with platform data logics. Informants’ calls for LMK digitalization indicate demand for accessible audit mechanisms that reduce reliance on unreadable reports and delayed distributions. Third, for labels/publishers and aggregators, the findings show the importance of flexible partnership arrangements that reduce harmful lock-in for senior artists with long catalogs and project-specific needs. The “layered contracts” described by KD require transparency support and clause education to reduce asymmetry in relationships.

Strengths and limitations. This study’s strength lies in its triangulated, mechanism-oriented design: narrative–biographical interviews with senior singers and industry stakeholders are complemented by systematic observation of public digital traces, enabling cross-validation of platform routines and governance constraints. The use of Atlas.ti-supported cross-case comparison and an audit trail strengthens interpretive transparency. However, the study is not statistically representative; no reliable population frame exists to estimate what proportion of Indonesia’s senior singers the participants represents. In addition, while genre and gender plausibly condition platform strategies, the sampling was not stratified systematically by these variables. Future research can extend this work through longer-term participant observation, genre-focused comparison (e.g., pop, dangdut, religious music, and traditional repertoires), and gender-sensitive analyses of how platform labour is distributed within teams and households (Xue, 2022; Yurui et al., 2024).

This study argues that Baby Boomer and Generation X singers do not experience a linear “shrinking of agency” in the platform era. What occurs is a reconfiguration of agency: a shift from dependence on older institutions toward more distributed work systems; a shift from unit-based measures of success toward data-based measures; and a shift from administrative copyright compliance toward compliance integrated into format design and channel selection. By linking empirical evidence (YNS, AD, IP, KD, RDS, VP) to structuration, platform rationalization, and AAA, the article shows that identity negotiation, algorithmic dynamics, and monetization practices operate as an interconnected sequence rather than separate domains. Layered strategic–practical–reflexive agency enables senior artists to sustain careers: maintaining algorithmic visibility while managing homogenization pressures, monetizing within rights constraints, and preserving rasa as a core source of artistic differentiation in an increasingly measured industry.

## Conclusion

This study argues that the agency of Baby Boomer and Generation X singers in Indonesia does not disappear under the dominance of algorithms, metrics, and copyright regimes. It is reconfigured into layered capacities—strategic, practical, and reflexive—that are continuously negotiated through three structuration modalities: signification, domination, and legitimation. Within signification, indicators of “success” shift from physical-sales certification toward cross-platform metrics that function as a new language for assessing position, performance, and strategic viability. Within domination, the scope of agency expands when artists consolidate digital, social, and symbolic resources through official channels as a “digital home,” access to aggregators/DSPs, and the insourcing of promotion and content operations to a core team or family members. Within legitimation, copyright compliance emerges as a strategic variable internalized in content design and channel selection (compliance by design), alongside demands for institutional reform through greater transparency and the digitalization of rights governance.

The study shows that rationalization and the tendency toward content homogenization in the platform economy do not automatically erase generational identity or diminish senior singers’ professionalism. Baby Boomers rearticulate the identity of “cassette/TV-era artists” into symbolic capital—as carriers of heritage and narrative depth—through storytelling formats, intimate performances, and repertoire continuity. Generation X, as a transitional cohort, manages a dual identity: maintaining professional authority while adopting more adaptive forms of digital communication, including internally managed promotion and asset consolidation on official channels. Under pressures of efficiency, calculability, predictability, and control, both generations negotiate boundaries so that “working with the algorithm” does not entail “abandoning rasa.”

The AAA framework appears as a set of concrete practices for income stabilization and expansion. Adaptation is visible in adjustments to format, narrative, and release calendars. Aggregation emerges through work standardization, consolidation of digital assets, and the formation of core teams that reduce transaction costs. Arbitrage appears through the use of differences across channels, timing, production costs, and rights regimes and catalog value. At the generational level, Generation X tends to apply format adaptation and channel arbitrage more assertively, while Baby Boomers show stronger emphasis on rights and reputation arbitrage, although both generations display a convergence process in practice.

The article’s main contribution is a layered agency model (strategic, practical, reflexive) that connects structuration theory, platform rationalization, and the AAA framework to interpret senior artists’ experiences in a Global South context. The findings show that career sustainability in the platform economy depends on more than technical skills. It depends on the capacity to build a work system (official channels, core teams, asset SOPs), interpret metrics as decision tools, and integrate rights compliance into strategic design. The platform-based music economy therefore introduces constraints for older generations while providing space to renegotiate identity, manage visibility, and develop a more diverse income portfolio that can withstand algorithmic change.

## Data Availability

The datasets generated and/or analyzed during the current study are not publicly available because they contain interview materials that may compromise participant confidentiality. De-identified excerpts supporting the conclusions are included in the article. Further information may be available from the corresponding author on reasonable request.
